# Three Release Rates of *Dicyphus hesperus* (Hemiptera: Miridae) for Management of *Bemisia tabaci* (Hemiptera: Aleyrodidae) on Greenhouse Tomato

**DOI:** 10.3390/insects10070213

**Published:** 2019-07-19

**Authors:** Hugh A. Smith, Karol L. Krey

**Affiliations:** 1 Gulf Coast Research and Education Center, University of Florida, Wimauma, FL 33598, USA; 2 USDA-ARS, 5230 Konnowac Pass Road, Wapato, WA 98951, USA

**Keywords:** sweetpotato whitefly, biological control, protected agriculture, zoophytophagous mirids

## Abstract

The sweetpotato whitefly, *Bemisia tabaci*, is a pest of greenhouse-grown tomato. Restrictions on insecticides in enclosed structures and the presence of commercial pollinators limit the options for the chemical control of whiteflies in greenhouses, increasing the importance of biological controls. *Dicyphus hesperus* is a zoophytophagous mirid predator native to North America. Three release rates of *D. hesperus* were evaluated on greenhouse tomato for control of the sweetpotato whitefly. The release rates were one, two or three adult *D. hesperus* per tomato plant each week for three weeks in cages containing four tomato plants and one mullein banker plant. There were fewer whitefly eggs in cages receiving predators than untreated cages one week after the third release, and fewer whitefly nymphs in cages receiving predators two weeks after the third release. There were no statistical differences in whitefly eggs or nymphs among predator release treatments. The highest release rate resulted in a 60% reduction in whitefly nymphs. Forty-two days after the first predator releases, there were no differences among release treatments in the number of *D. hesperus*. Our results indicate that *D. hesperus* can contribute management of *B. tabaci* on greenhouse tomato, but that it may be insufficient as a sole strategy.

## 1. Introduction

Florida is a leading producer of fresh market tomatoes in the United States, producing over $250 million of tomatoes in 2017 [1]. Tomatoes in Florida are primarily produced in the field, however the percentage of tomatoes and other vegetables produced in greenhouses and other protected structures has increased in recent years [2]. Greenhouse production provides the ability to grow indeterminate, closely spaced varieties that can produce higher yields over a longer period than determinate field grown varieties. Hochmuth and Toro [2] estimate that Florida greenhouse tomato production could yield $250,000 per acre. In addition, protected structures provide protection from extremes of low temperature and excessive precipitation, extending the season through the winter months in subtropical parts of the state. Protective structures provide an advantage compared to field production in pest management in that the migration of herbivores into the crop can be delayed. It is impossible to completely exclude pests from greenhouses, however, and after they have become established on the crop, the greenhouse may provide favorable conditions for pest population growth. One of the primary advantages of protected culture over field production is the improved environment for the release and establishment of biological control agents for pest management. Most varieties of tomato grown in greenhouses in Florida are pollinated using commercial hives. The presence of bees and restrictions on insecticides that can be applied in enclosed structures limit options for the chemical control of pests in protected structures, increasing the importance of biological control.

The sweetpotato whitefly, *Bemisia tabaci* MEAM1 (Gennadius), also known as the B biotype of *B. tabaci*, is a pest of many agronomic, ornamental, and horticultural crops, including tomato [3]. The sweetpotato whitefly transmits *Tomato yellow leaf curl virus* (TYLCV), and is the causal agent of irregular ripening of tomato, which is primarily associated with feeding by nymphs [4,5]. Biological control has been used with varying degrees of success to manage whiteflies in protected structures since the 1970s [6]. The parasitic wasps *Eretmocerus eremicus* Rose and Zolnerowich and *E. mundus* Mercet (Hymenoptera: Aphelinidae) have been used to manage *B. tabaci* in the United States [7,8]. In Europe, the predatory mirids *Macrolophus pygmaeus* Wagner and *Nesidiocoris tenuis* Rambur (Heteroptera: Miridae) are used effectively against whiteflies in greenhouse production [9]. Mirids are well-adapted to tomato and other solanaceous crops producing sticky exudates that impair other natural enemies [10]. Mirids deployed as biocontrol agents are zoophytophagous, sometimes feeding on plant tissue and capable of causing economic damage [11]. This has prevented the introduction of *M. pygmaeus* and *N. tenuis* into the Unites States for evaluation of their biological control potential [9]. The predatory mirid *Dicyphus hesperus* Knight is native to North America and has been evaluated for control of pests of greenhouse tomato in British Columbia since the late 1990s. McGregor et al. [12] determined that *D. hesperus* could complete its development feeding on nymphs of the greenhouse whitefly, *Trialeurodes vaporariorum* (Westwood), and that *D. hesperus* only fed on tomato fruits when foliage was absent [13]. Sanchez et al. [14] demonstrated that the presence of the banker plant mullein (*Verbascum thapsus* L.) increased the densities of *D. hesperus* in greenhouses compared to greenhouses that contained only tomato.

Promising results with *D. hesperus* for managing the greenhouse whitefly in temperate regions led us to evaluate the potential of managing sweetpotato whitefly under Florida’s subtropical greenhouse conditions. We were specifically interested in determining if *D. hesperus* could reduce densities of nymphs to below 0.5 nymphs per leaf, the threshold for development of irregular ripening of tomato [15], within two weeks of the initiation of releases. This two-week interval corresponds to the nymphal development timeframe for whiteflies on tomato under typical growing conditions [16]. Prompt reduction of nymphal densities by *D. hesperus* would indicate a role for the predator in reducing not only irregular ripening but also the secondary spread of TYLCV should it be introduced by viruliferous whiteflies into the greenhouse.

## 2. Materials and Methods

The efficacy of three release rates of *D. hesperus* for management of sweetpotato whitefly on greenhouse-grown tomato was evaluated and compared to an untreated control at the Gulf Coast Research and Education Center, Wimauma, FL, USA, in the fall of 2016. Tomato seeds (cv. Lanai) were planted mid Sept 2016 in 128 cell Speedling^®^ trays. Three weeks after emergence, seedlings were transplanted individually to 3.8-L pots filled with Fafard Professional Growing Mix. Mullein seedlings were purchased from Gloeckner and Company (550 Mamaroneck Avenue, Harrison, NY, USA) and were transplanted into 10-cm-diameter pots. Each mullein plant was provided with 20:20:20 water-soluble nutrient (10 g for 3 liters) in 125 mL water every 15 d until the initiation of the experiment. Four weeks after potting, four tomato and one mullein plant were placed into PVC frame cages, 80 cm in each dimension, enclosed in a white organdy cover (Jo Ann Fabric, University Park, FL, USA) that allowed air movement and light entry. Each cage comprised one experimental unit. Treatments were assigned to cages and arranged in a randomized complete block design with four replicates of each treatment. The cages were arranged on four greenhouse benches with each bench serving as a block. The plants were hand watered as needed and the tomatoes were fertilized with 45 g. of Osmocote^®^ Plus 15-9-12 fertilizer (The Scotts Company, Marysville, OH, USA) per pot every two weeks.

### 2.1. Plant Infestation

Cages were infested with 12 male and 12 female whitefly adults on 4 Nov and 50 of each sex on 15 Nov. The whiteflies were aspirated from a laboratory colony maintained at GCREC in organdy cages on cotton (*Gossypium hirsutum* L.) in a growth room at 30 °C (±2 °C), 50–70 % RH, and 14: 10 L: D. Whiteflies were aspirated into glass eyedroppers that were then examined under a stereomicroscope to confirm the number and gender in each vial prior to release into the cages.

### 2.2. Predator Releases

*Dicyphus hesperus* were shipped weekly for three weeks from Beneficial Insectary, Redding, CA, USA and released into cages at three treatment levels each week. Predators were released into treatment cages within 24 h of being delivered. *D. hesperus* were released into cages at densities of 4, 8 or 12 adults at 1:1 male:female ratio. Predators were released into cages on 22 and 29 Nov, and 6 Dec.

### 2.3. Sampling

One mid-stratum leaf with seven leaflets was removed from each cage weekly from 22 Nov to 27 Dec to assess predator impacts on the densities of whitefly eggs and nymphs. The 22 Nov sample was a pre-count taken immediately prior to the first introduction of predators into cages. Leaves were removed from one of the four plants in each cage each week, choosing a different plant each week and reinitiating the cycle on week five. Samples were taken to the laboratory where whitefly eggs and nymphs were counted on the lower leaf surfaces with the aid of a stereo microscope. Data were recorded as numbers of whitefly eggs and live whitefly nymphs. A final survey of all plants in each cage (four tomato plants and one mullein plant) was performed on 3 Jan 2017 to assess population levels of *D. hesperus* only. Temperatures inside two cages of the experiment were monitored with HOBO data loggers (Onset, Bourne, MA, USA).

### 2.4. Statistical Analysis

Statistics were conducted in R (version 3.4.1) [17]. Densities of whitefly eggs and nymphs were log transformed and compared with a repeated measures analysis of variance (ANOVA) using the lme function in the nlme package of R, with *Dicyphus hesperus* treatment (no, low, medium, high) and sample date (1–6) as predictor variables. Tukey’s post-hoc analysis was used to determine differences between groups using the glht function in the multcomp package of R. Densities of *D. hesperus* numbers were log transformed and analyzed with a mixed model ANOVA using treatment as a fixed effect with the lme function in the nlme package of R. Means were separated using Tukey’s HSD test (*p* < 0.05) in the agricolae package of R. In these models, we included random effects to control variation associated with sampling.

## 3. Results

Treatments and sample date had significant effects on whitefly egg numbers (treatment main effect: F = 18.34, df = 3, 9, *p* < 0.001; date main effect: F = 13.85, df = 3, 12, *p* < 0.001), and whitefly nymph numbers (treatment main effect: F = 9.39, df = 3, 9, *p* = 0.004; date main effect: F = 12.69, df = 3,12, *p* < 0.001).

**Figure 1:**
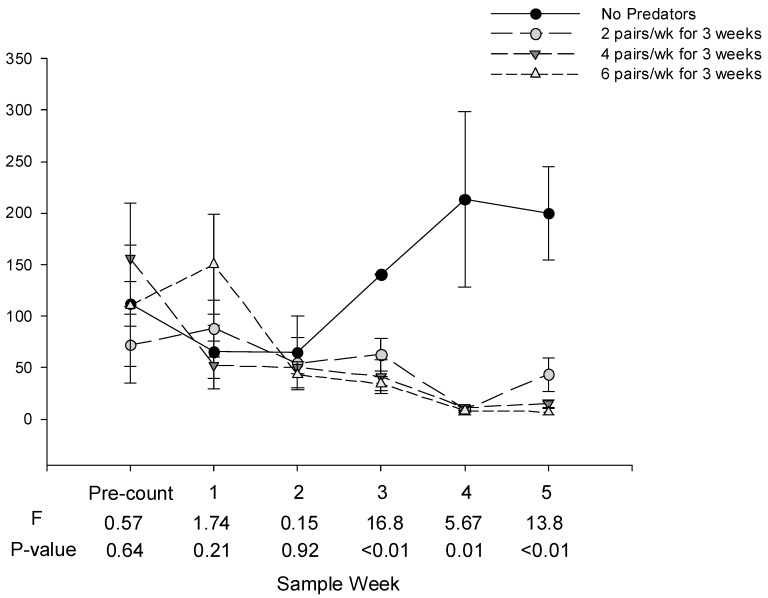
Mean (±SE) number of *B. tabaci* eggs per tomato leaf on tomato plants receiving low, medium and high release rates of *D. hesperus*. *D. hesperus* were released on 22 Nov, when the pre-count was taken, and 29 Nov and 6 Dec, 2016, the same days that readings 1 and 2 were collected; when sample dates coincide with release, cages were sampled first.

**Figure 2:**
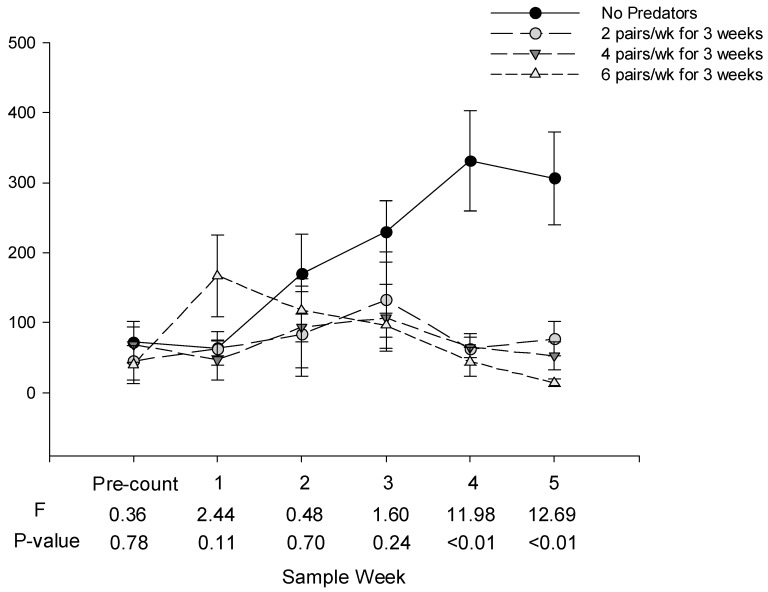
Mean (±SE) number of *B. tabaci* nymphs per tomato leaf on tomato plants receiving low, medium and high release rates of *D. hesperus. D. hesperus* were released on 22 Nov, when the pre-count was taken, and 29 Nov and 6 Dec, 2016, the same days that readings 1 and 2 were collected; when sample dates coincide with release, cages were sampled first.

There were no statistical differences in the numbers of whitefly eggs or nymphs among treatments in the pre-count or one week after the first and second predator releases (Sample weeks 1 and 2, Figure 1 and Figure 2). One week after the third release, there were significantly fewer eggs in the cages receiving predators than in cages not receiving predators. There were significantly fewer eggs and nymphs in cages receiving predators than in cages not receiving predators four and five weeks after the third release. There were no statistical differences in the numbers of whitefly eggs or nymphs among cages receiving high, medium or low numbers of predators on any date.

There were significantly more second instar *D. hesperus* nymphs in cages receiving high numbers of predators when cages were broken down 42 d after the first release (Table 1). However, numbers of other *D. hesperus* life stages were not statistically different across treatments receiving predators and overall, the total number of *D. hesperus* nymphs and adults was not different among predator treatments. No *D. hesperus* were recovered from the control cages. Weekly temperatures averaged 21.4 °C, with weekly maximums and minimums averaging 27.2 °C and 16.6 °C.

## 4. Discussion

Other studies evaluating the ability of *D. hesperus* to suppress *B. tabaci* on greenhouse tomato used repeated low-level releases of prey over a period of several weeks, and initiated releases of both prey and predator at the outset of the trials [9,18]. Calvo et al. [9] observed that densities of whitefly nymphs became higher on plants without predators eight weeks after initial releases during a fall experiment, and 12 weeks in a summer experiment. Calvo et al. [18] reported that *B. tabaci* nymph densities became significantly higher in the absence of *D. hesperus* only 17 weeks after low level weekly releases of predator and prey. In these trials, *Ephestia kuhniella* eggs were provided during the first weeks as a supplementary protein source for *D. hesperus* nymphs, because the prey was assumed to be too scarce to allow *D. hesperus* nymphs to reach the adult stage [19]. A similar approach was taken for the evaluation of the predatory mirid *Nesidiocoris tenuis* (Reuter) for management of *B. tabaci* in greenhouse tomato [20]. *Ephestia kuhniella* eggs were provided and whiteflies were released at a rate of ten adults per plant for three weeks. In that trial, there were no differences in whitefly nymph numbers between plants receiving *N. tenuis* and plants without predators until 10 weeks after the initial releases. Releases of two *N. tenuis* per plant reduced whitefly nymph numbers to two to three per plant after 13 weeks. To evaluate *D. hesperus* for control of the greenhouse whitefly, *Trialeurodes vaporariorum* Westwood, on greenhouse tomato where mullein was present, Sanchez et al. [14] released 1.25 whitefly adults per plant followed by 1.25 *D. hesperus* adults per plant three weeks later. Whitefly nymphs peaked at 26.5 to 35 nymphs per tomato leaf about 90 days after the initial whitefly release and 70 days after the *D. hesperus* release, after which *D. hesperus* brought the nymph densities down to sub-economic levels.

In the trials referenced above, both whitefly prey and mirid predator developed through at least two generations on tomato before the predator reduced prey densities, and in trials with *B. tabaci*, the additional step of supplying *E. kuhniella* eggs was introduced. Our objective was to collect data on release rates of *D. hesperus* needed to reduce whitefly nymph densities promptly and with a minimum of additional management requirements for the grower. MacGregor et al. [12] demonstrated that *D. hesperus* females can consume approximately 24 young *T. vaporariorum* nymphs a day, and Sanchez et al. [19] estimated that *D. hesperus* females could live for seven weeks on tomato when provided with a protein source. Based on predation rates observed for *D. hesperus* on *T. vaporariorum* nymphs, we assumed three releases of low numbers of *D. hesperus* adults would reduce whitefly nymph densities on a moderately infested tomato plant to a few nymphs per leaf a few weeks after the third release. We suspect that the primary reason our results differed from those of previous studies is that we allowed whitefly egg and nymph densities to develop to significantly higher densities before initiating predator releases. In addition, we used a more confined arena than other studies, which employed sections of greenhouses and allowed *D. hesperus* to move from plant to plant. Our approach did not recreate actual greenhouse growing conditions as closely as other studies but should have provided *D. hesperus* optimal conditions to demonstrate its predatory capacity because predators were confined to four tomato plants and had access to mullein, which has been demonstrated to improve establishment of the predator [14].

While the studies outlined above were considered successful examples of suppression of whitefly populations by *D. hesperus* from a research perspective, the long-term presence of whitefly populations and the peak pest densities achieved in some studies could produce crop damage that might not be tolerable to some growers. A major concern of growers of greenhouse tomato in Florida is the introduction of TYLCV by viruliferous whiteflies. The virus can be transmitted in 15 m or less of feeding [21]. Biocontrol agents do not work quickly enough to reduce primary transmission of the virus, which is caused by viruliferous whiteflies migrating into the greenhouse. Ideally, a biocontrol agent for the management of *B. tabaci* and TYLCV on greenhouse tomato could contribute to the reduction of secondary transmission of the virus. Secondary transmission is caused by whiteflies that develop on infected plants in the greenhouse, move to healthy plants as adults, and transmit the virus to them. A biocontrol agent that could significantly reduce numbers of whitefly nymphs before they emerge as adults would contribute to reductions in secondary transmission of TYLCV. The levels of predation observed by *D. hesperus* in *T. vaporariorum* suggested that *D. hesperus* might be capable of reducing whitefly nymphal populations during the time period needed to develop from crawler to adult (14–15 d at 25 °C) [16]. The results of our trial and of other trials discussed above do not indicate that *D. hesperus* releases significantly impacted the first generation of whitefly nymphs to which they were exposed. Whitefly nymph numbers were not reduced to economically acceptable levels even in the high release rate three weeks after the third release. The average whitefly nymph numbers in the final reading of the high release rate were 15 nymphs per leaflet. Thirty-six *D. hesperus* adults released on four tomato plants over a three-week period reduced the pre-count whitefly nymph infestation of 40 nymphs/per leaf by only 63 percent, and only after it had more than doubled during the interim weeks. The threshold to apply insecticides to manage irregular ripening of tomato is five whitefly nymphs per 10 leaflets or 0.5 whitefly nymphs per leaflet [15]. Fifteen nymphs per 7 leaflets averages 2.1 nymphs per leaflet, which significantly surpasses the threshold for treatment for irregular ripening.

Efforts to improve the reduction of whiteflies by *D. hesperus* on greenhouse tomato by adding other biocontrol agents have been met with limited success. Bennet et al. [22] found that releasing *D. hesperus* in combination with the parasitoid *Encarsia formosa* Gahan (Hymenoptera: Aphelinidae) for the management of *T. vaporariorum* did not enhance suppression of the whitefly. Calvo et al. [18] found that releasing *Eretmocerus eremicus* Rose & Zolnerowich (Hymenoptera: Aphelinidae) with *D. hesperus* increased suppression of *B. tabaci* nymphs on greenhouse tomato by only nine percent, 13 weeks after release of biocontrol agents. A future avenue of research could evaluate the compatibility of *D. hesperus* with biopesticides, including insecticidal soaps, which have demonstrated efficacy against *B. tabaci* on tomato under greenhouse conditions [23]. In addition, there have been significant advances in the development of tomato varieties with tolerance to TYLCV [24,25]. Virus-tolerant tomato varieties that can accommodate higher levels of *B. tabaci* may provide broader opportunities for the integration of biocontrol agents such as *D. hesperus* into greenhouse pest management programs.

## 5. Conclusions

The results of our trial are consistent with other studies demonstrating that *D. hesperus* can reduce numbers of *B. tabaci* on greenhouse tomato, but that the number of weeks required to bring whiteflies to sub economic levels may not be considered practical by greenhouse growers. *Dicyphus hesperus* can clearly contribute to management of *B. tabaci* on greenhouse tomato if introduced early when whitefly populations are low, but additional research is needed to determine the optimal timing and rates of *D. hesperus* releases within a season-long management plan for the pest.

## Figures and Tables

**Table 1:** Mean (±SE) number of nymphal and adult *D. hesperus* 42 days after the initial release in cages containing four tomato and one mullein plant infested with *B. tabaci*.

No. *D. hesperus* Pairs Released per Week for 3 Weeks *^a^*	First Instar	Second Instar	Third Instar	Fourth Instar	Adult	Total
0	0.0 ± 0.0b	0.0 ± 0.0c	0.0 ± 0.0b	0.0 ± 0.0b	0.0 ± 0.0b	0.0 ± 0.0b
2	15.8 ± 1.3a	42.5 ± 7.0b	34.0 ± 9.0a	21.8 ± 5.5ab	26.0 ± 5.1a	140.0 ± 12.7a
4	25.5 ± 3.3a	44.0 ± 4.2b	38.3 ± 8.2a	42.8 ± 18.1a	25.3 ± 9.5a	175.8 ± 29.2a
6	25.0 ± 6.2a	67.3 ± 2.3a	32.0 ± 3.9a	14.0 ± 3.3ab	19.0 ± 4.5ab	157.3 ± 5.2a
F _3,9_	11.39	44.24	7.56	3.46	4.29	24.66
*p*-value	0.0008	<0.0001	0.004	0.051	0.029	<0.0001

Each cage contained four tomato plants and one mullein plant. Means within a column followed by the same letter are not significantly different by Tukey’s Studentized Range test (*α* = 0.05). ***^a^*** Two, four or six pairs of male and female *D. hesperus* were released in designated cages on 22 and 29 Nov and 6 Dec 2019.
